# Chromatin determinants of the inner-centromere rely on replication factors with functions that impart cohesion

**DOI:** 10.18632/oncotarget.11982

**Published:** 2016-09-12

**Authors:** Takuya Abe, Ryotaro Kawasumi, Hiroshi Arakawa, Tetsuya Hori, Katsuhiko Shirahige, Ana Losada, Tatsuo Fukagawa, Dana Branzei

**Affiliations:** ^1^ IFOM, The FIRC Institute for Molecular Oncology Foundation, Milan, Italy; ^2^ Department of Chemistry, Graduate School of Science and Engineering, Tokyo Metropolitan University, Minamiosawa, Hachioji-shi, Tokyo, Japan; ^3^ Graduate School of Frontier Biosciences, Osaka University, Suita, Osaka, Japan; ^4^ Laboratory of Genome Structure and Function, Research Center for Epigenetic Disease, Institute of Molecular and Cellular Biosciences, University of Tokyo, Yayoi Bunkyo-Ku, Tokyo, Japan; ^5^ Chromosome Dynamics Group, Molecular Oncology Program, Spanish National Cancer Research Centre, Madrid, Spain

**Keywords:** sister chromatid cohesion, inner-centromere, replication stress, DDX11, Tim-Tipin, Chromosome Section

## Abstract

Replication fork-associated factors promote genome integrity and protect against cancer. Mutations in the DDX11 helicase and the ESCO2 acetyltransferase also cause related developmental disorders classified as cohesinopathies. Here we generated vertebrate model cell lines of these disorders and cohesinopathies-related genes. We found that vertebrate DDX11 and Tim-Tipin are individually needed to compensate for ESCO2 loss in chromosome segregation, with DDX11 also playing complementary roles with ESCO2 in centromeric cohesion. Our study reveals that overt centromeric cohesion loss does not necessarily precede chromosome missegregation, while both these problems correlate with, and possibly originate from, inner-centromere defects involving reduced phosphorylation of histone H3T3 (pH3T3) in the region. Interestingly, the mitotic pH3T3 mark was defective in all analyzed replication-related mutants with functions in cohesion. The results pinpoint mitotic pH3T3 as a postreplicative chromatin mark that is sensitive to replication stress and conducts with different kinetics to robust centromeric cohesion and correct chromosome segregation.

## INTRODUCTION

Various stressful conditions and difficult-to-replicate regions encountered during DNA replication need specialized replication factors to preserve genome stability. Some replication fork components play multiple roles in ensuring genome stability and are required to integrate various responses, such as topological transitions, checkpoint activation, chromatin assembly and sister chromatid cohesion (SCC) [1, 2]. Examples of factors with variegated roles during DNA replication include the replication fork protection complex, composed of Tim (also known as Timeless) and Tipin [3], and the DDX11/ChlR1 helicase [4]. These factors interact with each other and jointly affect SCC in human cells [5, 6].

SCC is a prerequisite for accurate chromosome segregation and is established during DNA replication by cohesin and regulatory factors [7]. The cohesin core, composed by Smc1, Smc3 and Scc1 (also known as Mcd1 and Rad21), is loaded onto chromatin by Scc2/Scc4 prior to replication, and holds DNA strands within its ring structure. Scc1 also binds to the fourth cohesin core subunit, which is known as Scc3 or stromal antigen, present as two variants in vertebrates, SA1 and SA2. Rad21 and SA proteins further associate with several other factors, including Pds5, Sororin, and Wapl, which enable cohesion establishment, maintenance and dissolution during cell cycle progression [7].

Establishment of cohesion requires Smc3 acetylation by the acetyltransferase Eco1 in budding yeast and its vertebrate orthologues, ESCO1 and ESCO2 [8, 9]. Smc3 acetylation occurs during the progression of DNA replication in both yeast and human, probably by Eco1 recruitment to replication forks via its interaction with the polymerase clamp, PCNA [10]. Smc3 acetylation, together with its interaction with Sororin in mammalian cells [11], causes an increased residence time of cohesin on chromosomes, leading to improved cohesion [7]. Cohesion establishment is generally coupled to DNA replication, and numerous replication fork components that safeguard genome integrity are involved in this process [7].

Cohesion factors that are not part of the cohesin ring play crucial roles in genome integrity [12], but their function in cohesion establishment/maintenance remains hardly understood. Mutants in many replication fork-associated cohesion factors show reduced acetylated Smc3 in budding yeast [13-15]. This observation can be explained by a multitude of mechanisms, including defects in cell cycle, altered residence time of cohesin on chromatin, and/or reduced accessibility of the Eco1 acetyltransferase to cohesin. Recent work indicates that the cohesion defects as well as other DNA damage tolerance problems associated with cohesion mutants with roles in DNA replication may be a secondary effect of persisting replication-associated DNA lesions and/or altered replication fork topology [16]. However, the relationship between replication lesions and cohesion remains to date elusive.

Mutations in some of replication-fork associated cohesion factors cause human developmental disorders known as “cohesinopathies” [17]. Roberts syndrome/SC phocomelia (RBS), and Warsaw Breakage syndrome (WABS) are two recessive cohesinopathies, caused by homozygous mutations in single genes, *ESCO2* and *DDX11/CHLR1*, respectively [8, 18, 19]. DDX11 is an evolutionarily conserved superfamily 2 iron-sulfur cluster DEAH-box DNA helicase homologous with budding yeast Chl1 [4], and its functions in the context of SCC are likely important for development [20]. Budding yeast Eco1 and Chl1 physically interact, and *CHL1* deletion is synthetic lethal with temperature sensitive *eco1* alleles [13, 21]. However, insights in the functional relationship between vertebrate Eco1/ESCO2 and Chl1/DDX11 are currently lacking.

Here, we used genetically amenable chicken lymphoma DT40 cell lines that have a stable karyotype to establish model cell lines of WABS and RBS, as well as a combination of these mutations and their related genes. Our results unveil essential complementarity between vertebrate ESCO2 and either DDX11 or Tipin in relation to chromosome segregation and proliferation, and between DDX11 and ESCO2 in centromeric cohesion. Importantly, we uncover that even mild defects in replication-mediated cohesion invariably associate with reduced concentration of histone H3T3 phosphorylation at the centromere. We further show that additional cohesion mutations in complementary pathways act as enhancers to push replication cohesion mutants to death by exacerbating the inner-centromere dysfunction and causing chromosome missegregation, accompanied or not by visible centromeric cohesion defects. In conclusion, our findings pinpoint the mitotic pH3T3 at the inner-centromere as a good indicator of cohesion insufficiency and reveal the kinetics by which certain forms of replication stress negatively influence chromosome stability in mitosis.

## RESULTS

### Establishment of WABS and RBS model cell lines in DT40

**Figure 1:**
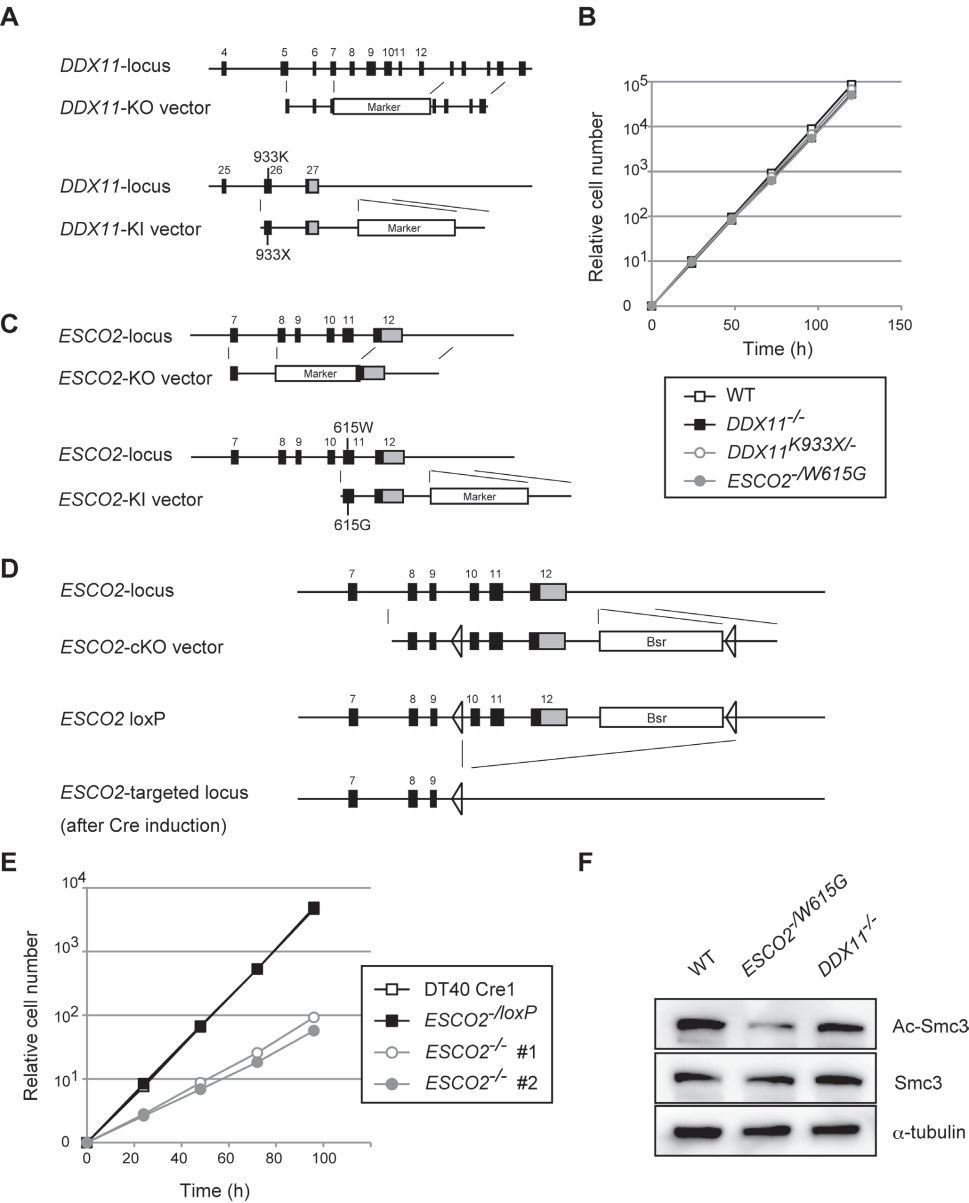
Establishment and general characterization of WABS- and RBS-model DT40 lines **A.** Schematic representation of gene-targeting KO and KI constructs for *DDX11*. Black-filled rectangles indicate exons, and “Marker” indicates drug resistance genes. **B.**, **E.** Growth curves. **C.**-**D.** Schematic representation of KO and KI constructs (C) and conditional gene-targeting constructs (D) for *ESCO2*. Black-filled rectangles and Bsr indicate exons and the Blasticidin S-resistance gene, respectively. **F.** Western blotting from total cell lysates for the markers indicated. The results were confirmed with lysates from an independent biological experiment.

To establish WABS model cell lines in DT40, we designed a DDX11 knock-out (KO) construct that deletes exons 7 to 12, and a knock-in (KI) construct that introduces a deletion mutation of K933 (hereby referred to as K933X) in the very C-terminus of DDX11 (Figure 1A). K933 corresponds to K897 of human DDX11 (hDDX11), found in the first identified WABS patient [18]. As WABS is a recessive disorder caused by inactivation of both alleles [18, 19], we generated *DDX11*^−/−^ as well as *DDX11^K933X/−^* cell lines. We verified the correct establishment of these strains by reverse transcription polymerase chain reaction (RT-PCR) and genomic sequencing (Supplementary Figures S1A-B). Growth curves revealed similar proliferation rates of *DDX11*^−/−^ and *DDX11^K933X/−^* cells with wild-type (WT) (Figure 1B). Thus, DDX11 is not critical for proliferation in somatic cells, although it is essential for development [20].

Several RBS patients carry the W539G mutation in the acetyltransferase catalytic domain of human ESCO2 (hESCO2) on one allele, and truncating mutations on the other [8, 22]. We generated KO and KI constructs for the chicken *ESCO2* (*cESCO2*) gene that disrupt exons 8 to 11 of *cESCO2* and introduce the W615G mutation in cESCO2, corresponding to the hESCO2 W539G mutation, respectively (Figure 1C). We next established *ESCO2^−/W615G^* cells and confirmed the genotype by RT-PCR and genomic sequencing (Supplementary Figure S1C-D).

To construct conditional *ESCO2* KO cells, here referred to as *ESCO2*^−/loxP^, we sequentially transfected the *ESCO2* KO construct and a conditional KO (cKO) construct that introduces two LoxP sites into the *ESCO2* gene locus (Figure 1D) to DT40 Cre1 cells (Table 1), which stably express the Cre recombinase [23] (note that the growth curves of DT40 Cre1 WT cells overlap with the ones of *ESCO2*^−/loxP^). Subsequently, we activated the Cre recombinase by addition of 4-Hydroxytamoxifen and obtained *ESCO2*^−/−^ cell lines. *ESCO2^−/W615G^* cells proliferated similarly with WT and *DDX11* mutant cells (Figure 1B), while *ESCO2*^−/−^ cells were viable but showed severe proliferation defects (Figure 1E). These results indicate that ESCO2 is important for cellular proliferation, but the *cESCO2-W615G* mutation supports normal proliferation.

Because both ESCO1 and ESCO2 contribute to Smc3 acetylation at K105 and K106 residues [7, 9], and this is critical for cohesion by stabilizing cohesin on chromatin [11], we next monitored acetylated Smc3 (Ac-Smc3) in the generated *ESCO2* and *DDX11* mutants. *DDX11*^−/−^ did not cause a marked defect in Ac-Smc3, which was however decreased in *ESCO2^−/W615G^* (Figure 1F), to similar levels as in *ESCO2*^−/−^ cells (Supplementary Figure S1 E). Thus *ESCO2^−/W615G^* mutation is hypomorphic in regard to Smc3 acetylation (see also below), but sustains normal proliferation.

### Cohesion deficiency in DT40 models of RBS and WABS

**Figure 2:**
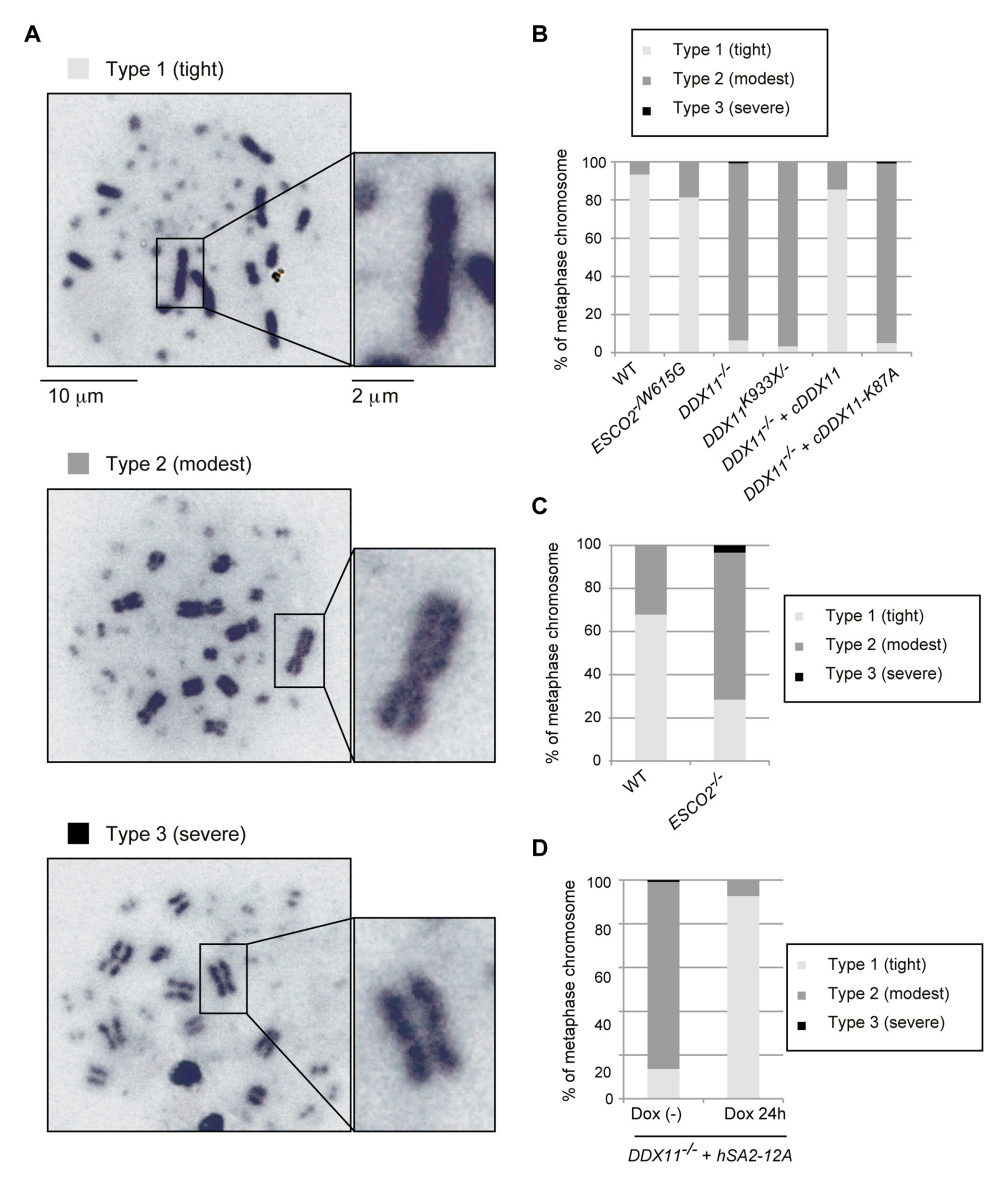
Cohesion defects in various *DDX11* and *ESCO2* mutants **A.**-**D.** Chromosomes from metaphase spreads were classified in three groups (A), and more than 100 metaphase cells were analyzed for each genotype (B-D). Independently prepared slides, from a different biological experiment, were used to confirm the trend. (D) *DDX11* cells with *hSA2-12A* were incubated with or without Dox, to induce expression of *hSA2-12A*, for 24 h and metaphase spread samples were examined as in (B-C).

We next examined metaphase spreads of *ESCO2^−/W615G^*, *DDX11*^−/−^ and *DDX11^K933X/−^* cells for cohesion defects. In DT40 WT cells, most of metaphase cells have typically tight chromosomes, with the sister chromatids being in close proximity also on chromosome arms (Figure 2A, Type 1, fully cohered). Only 5-25% of metaphase cells have X-shaped chromosomes, in which the sister chromatids are united at the centromeric regions, but the arms are apart (Figure 2A, Type 2) [24-26]. Severely centromeric cohesion-defective chromosomes (Figure 2A, Type 3) are not observed in WT cells. These features of DT40 cells allow identification of cohesion defects limited to chromosome arms, which are not visible in metaphase spreads of human cell lines due to a strong prophase pathway of sister chromatid resolution causing opening of the chromosome arms [27].

*ESCO2^−/W615G^* cells had a karyotype distribution similar to WT cells (Figure 2B), while *ESCO2*^−/−^ mutation caused a strong increase in cells with arm cohesion defects. Moreover, a small percentage of *ESCO2*^−/−^cells had severe premature centromeric separation (Figure 2C). These results substantiate the notion that *ESCO2^−/W615G^* is only mildly defective in ESCO2 functions (see below). Since *ESCO2*^−/−^ cells have severe proliferation defects (Figure 1E), in the following, we used *ESCO2^−/W615G^* as RBS model cell line.

Most *DDX11*^−/−^ and *DDX11^K933X/−^* metaphase cells showed arm cohesion defects (Figure 2B). To address whether the SCC defects of *DDX11*^−/−^ cells relate to DDX11 helicase activity deficiency, we expressed WT *cDDX11* or helicase-dead *cDDX11*-K87A, in which a critical lysine residue in the Walker A motif of DDX11 was substituted by alanine. Exogenously expressed cDDX11 rescued the SCC defects of *DDX11*^−/−^ cells, but *cDDX11*-K87A did not (Figure 2B), indicating that the helicase activity is important for DDX11 function in SCC.

In vertebrates, cohesin removal from chromosome arms in mitotic prophase involves Plk1-dependent phosphorylation of the SA2 subunit of cohesin [27]. Because Smc3 acetylation, which is important for cohesion establishment, did not appear severely defective in *DDX11*^−/−^ cells (Figure 1F), we examined if preventing the removal of cohesin from chromosome arms would suppress the observed SCC defects of *DDX11*^−/−^. Expression of the hSA2-12A variant, which is resistant to the Plk1-mediated phosphorylation, caused increased retention of cohesin on metaphase chromosomes ([27] and Supplementary Figure S2A), and suppressed the cohesion defects of *DDX11*^−/−^ cells (Figure 2D). We note that, unlike expression of hSA2-12A, expression of hSA2 did not improve the cohesion defects of *DDX11*^−/−^ cells (Supplementary Figure S2A), although the analyzed cells expressed similar levels of hSA2 and hSA2-12A (Supplementary Figure S2B). Together, the results indicate that vertebrate DDX11 is dispensable for Smc3 acetylation, but significantly contributes to SCC establishment/maintenance on chromosome arms.

### Combination of WABS and RBS mutations causes synthetic lethality

**Figure 3:**
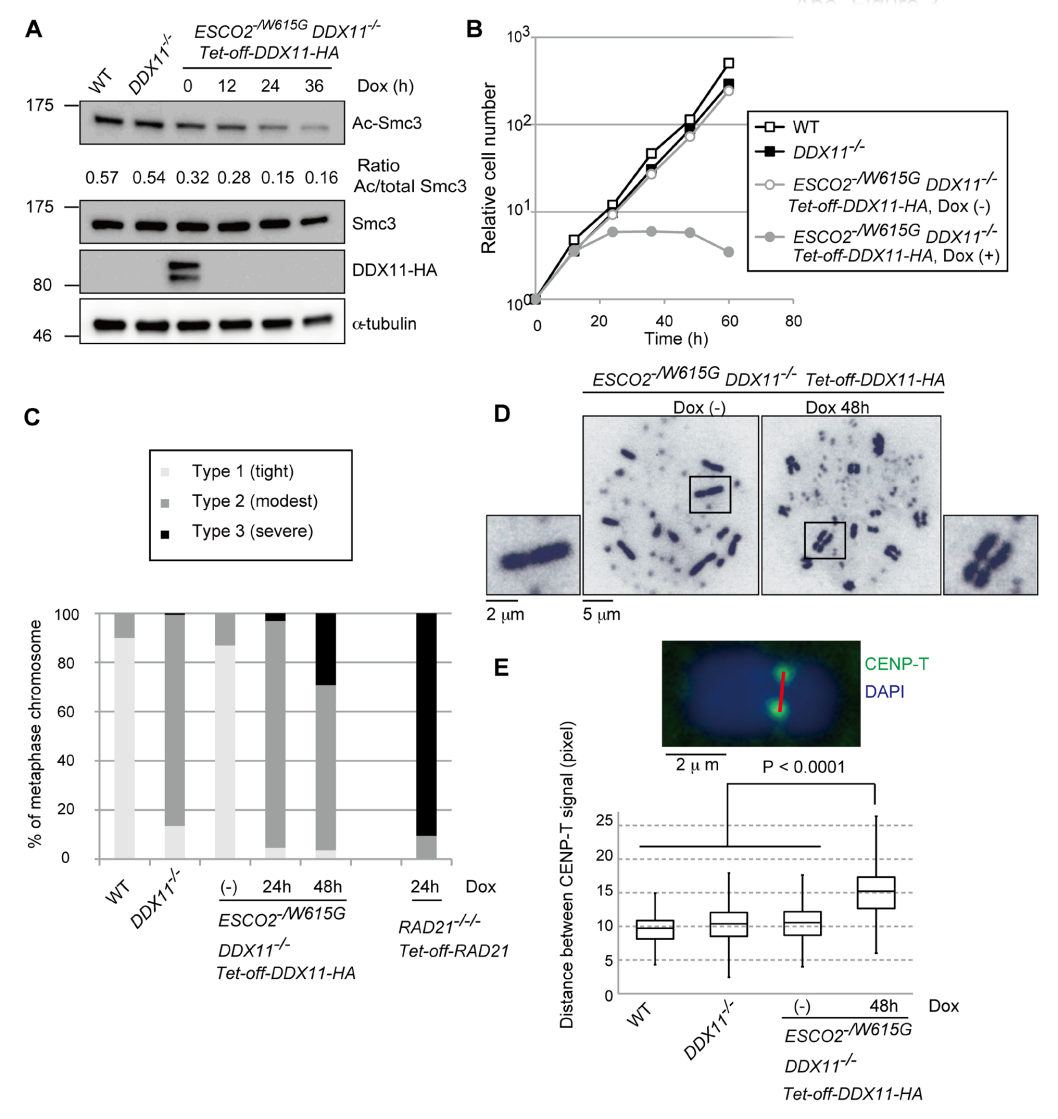
Synthetic lethality between *DDX11*^−/−^ and *ESCO2^−/W615G^* mutations **A.** Depletion of DDX11-HA protein and measurement of Ac-Smc3, Smc3, and α-tubulin (loading control) by Western blotting. The results were confirmed with lysates from an independent biological experiment. **B.** Growth curves. Dox was added at time 0. **C.**-**D.** Metaphase spreads examined as in Figure 2B with more than 100 metaphases examined for each genotype and independently prepared slides, from a different biological experiment, used to confirm the trend. **E.** Metaphase spread samples were prepared by the cytospin method after incubation with 0.1 μg/ml of colcemid for 1 h, and metaphase spread samples were prepared by the cytospin method. The distances between CENP-T signals were measured for more than 275 chromosomes. The same trend was confirmed from an independent biological experiment. *p* values were calculated by Student's t-test.

To examine the genetic relationship between causal mutations of WABS and RBS, we next generated conditional *ESCO2^−/W615G^ DDX11*^−/−^ double mutants. In brief, we introduced in *ESCO2^−/W615G^ DDX11^−/+^* cells the tetracycline transactivator (tTA) system and a *cDDX11-HA* construct, wherein *cDDX11-HA* expression can be repressed by addition of Doxycycline (Dox) (Supplementary Figure S3A). Hereafter we refer to this system as *Tet-off-DDX11*-HA. We next disrupted the second allele of *DDX11*. After Dox addition, the detectable level of DDX11-HA disappeared within 12 hours and Ac-Smc3 levels also decreased (Figure 3A), probably because of the reduced percentage of cells in S phase (Supplementary Figure S3B). The proliferation of *ESCO2^−/W615G^ DDX11*^−/−^ cells was severely impaired, with cells stopping to proliferate starting at 24 hours after Dox addition (Figure 3B). These cells also displayed a prominent accumulation in sub-G1 (Supplementary Figure S3B), indicative of cell death. Notably, *ESCO2^−/W615G^ DDX11*^−/−^ cells expressing the *Tet-off-DDX11-HA* construct showed predominantly modest cohesion defects 24 hr after Dox addition, while at 48 hr following Dox addition, more than 20% of cells showed severe centromeric cohesion defects (Figure 3C-3D). Although this percentage is lower than in *RAD21* conditional KO cells ([24] and Figure 3C), the results reveal that the cohesion defects of *ESCO2^−/W615G^* cells at centromeres are masked in the presence of functional DDX11, and vice-versa.

As only double *ESCO2^−/W615G^ DDX11*^−/−^ mutants, but not individual single mutants, exhibited metaphase cells with premature centromeric sister chromatid separation (Figure 3C), the results indicate that ESCO2 and DDX11 compensate for each other in engendering robust cohesion in this region. In line with the above-mentioned result, distances between sister chromatids at centromeric regions (marked by CENP-T) were significantly increased in *ESCO2^−/W615G^ DDX11*^−/−^ cells in comparison with single mutants and WT (Figure 3E). Thus, ESCO2 and DDX11 act complementarily to enable robust sister chromatid cohesion at centromeres, and concomitant dysfunction of these proteins causes lethality.

### Missegregating chromosomes and centromeric cohesion defects in *ESCO2^−/W615G^ DDX11*^−/−^ cells

**Figure 4:**
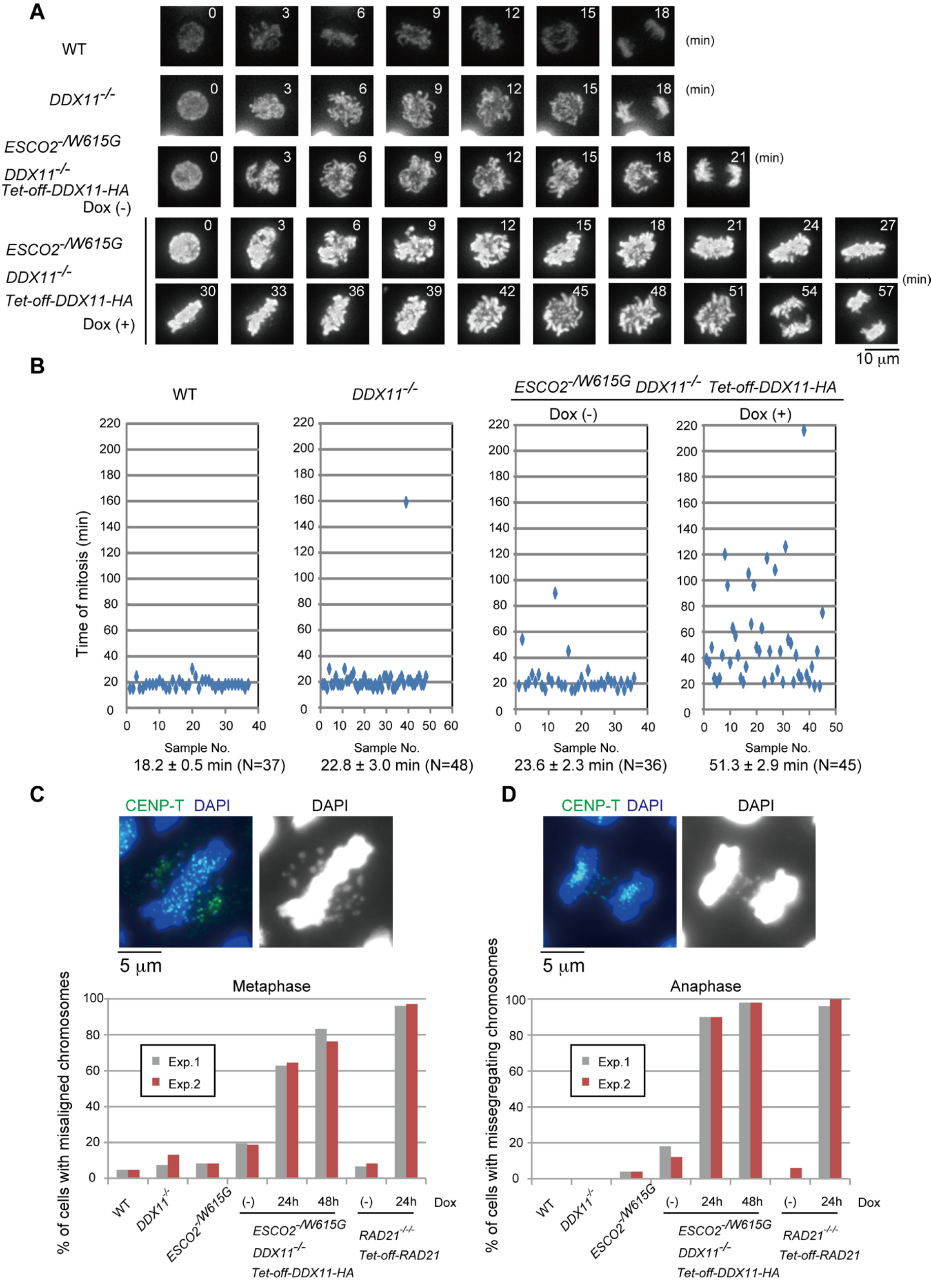
*ESCO2^−/W615G^ DDX11*^−/−^ cells show mitotic delays and chromosome missegregation **A.** Dynamics of mitotic chromosomes in the indicated cell lines. Live cell imaging was initiated 24 h after Dox addition and continued until 32 h. **B.** Quantification of the time for progression from prophase to telophase. N represents the number of cells examined, the average time required to complete mitosis is indicated under the panels. **C.**-**D.** Misaligned chromosomes in metaphase (C) or missegregating chromosomes in anaphase (D). At least 100 cells for the metaphase plot and 50 cells for the anaphase plot were analyzed for each experiment. The results of two independent experiments are plotted.

To visualize chromosome behavior during mitotic progression, we expressed histone H2B-mCherry in all relevant cell lines (Figure 4A). Whereas both *DDX11*^−/−^ and *ESCO2^−/W615G^* cells (*ESCO2^−/W615G^ DDX11*^−/−^ cells complemented with *cDDX11*)** showed mild mitotic delays, *ESCO2^−/W615G^ DDX11*^−/−^ cells had very long mitoses, with the delay being particularly severe at the metaphase to anaphase transition (Figure 4A-4B). In line with the above-mentioned results, we found an increase in the percentage of metaphase cells in *ESCO2^−/W615G^ DDX11*^−/−^ compared to control cell lines, but a similar percentage of anaphase cells (Supplementary Figure S4).

Next, we examined chromosome alignment and segregation in asynchronous cells of the relevant genotypes. Similarly to conditional *RAD21^−/−/−^*, kept alive with a *Tet-off-RAD21* construct [24], *ESCO2^−/W615G^ DDX11*^−/−^ cells** had a high percentage of misaligned chromosomes in (pro)metaphase (Figure 4C), and more than 90% of *ESCO2^−/W615G^ DDX11*^−/−^ had missegregating chromosomes at anaphase (Figure 4D). This finding indicates that the anaphase is highly catastrophic in *ESCO2^−/W615G^ DDX11*^−/−^, thus exposing a potential cause of lethality.

Interestingly, we noticed that 24 hr after Dox addition, *ESCO2^−/W615G^ DDX11*^−/−^ cells did not show severe cohesion defects (Figure 3C), but had rampant chromosome missegregation, in ranges similar to conditional *RAD21^−/−/−^* cells (Figure 4C-4D), which however were highly defective in centromeric cohesion as well (Figure 3C and [24]). Thus, chromosome missegregation happens in *ESCO2^−/W615G^ DDX11*^−/−^ cells before premature sister chromatid separation at centromeres can be observed cytogenetically. This result made us speculate that both the overt premature centromeric sister chromatid separation and chromosome missegregation phenotypes are associated with, and possibly caused by a centromeric defect not visible by classically employed cytogenetic approaches.

### *ESCO2* and *DDX11*-deficient cells show diffused inner centromere H3T3 phosphorylation and Aurora B mislocalization

**Figure 5:**
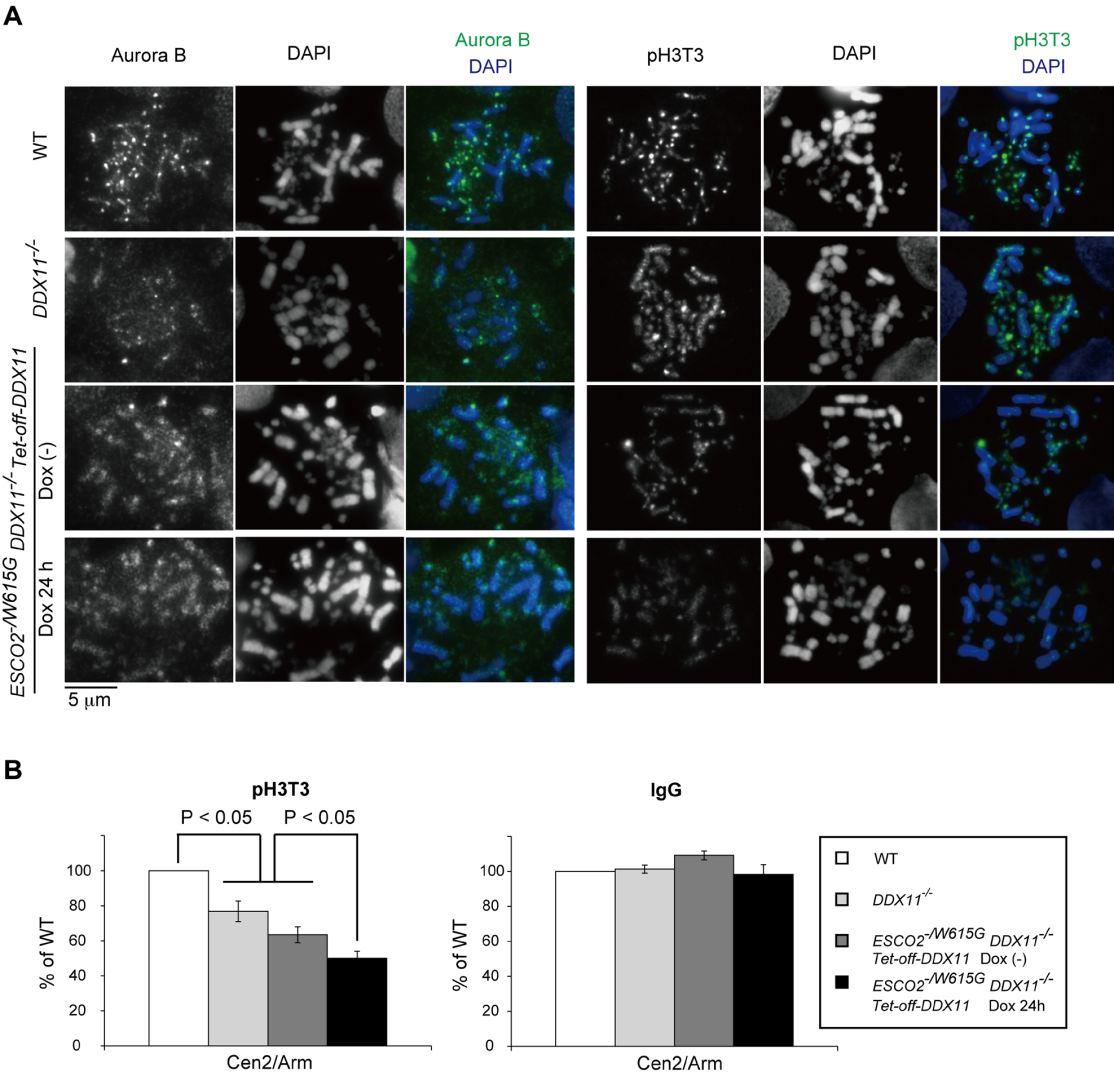
*ESCO2^−/W615G^ DDX11*^−/−^ cells show inner centromere structural defects **A.** Localization of Aurora B and phosphorylated-Histone H3T3 (pH3T3) from samples prepared as in Figure 3E. **B.** ChIP-qPCR of pH3T3 at a centromeric (CEN) region. The ratios of pH3T3 level between Cen2 and MHM repeats (Arm) were normalized by those of Input samples. Simultaneously immunoprecipitated DNAs with control IgG antibody and anti-pH3T3-coupled beads were amplified, and the ratios were calculated in the same way. Specific amplification of the centromeric regions and MHM repeats was controlled by PCR. Experiments were repeated three times in independent biological experiments. Bars indicate standard deviations. *p* values were calculated by Student's t-test.

Because of the cohesion and mitotic defects** of *ESCO2^−/W615G^ DDX11*^−/−^ cells, we asked if the inner-centromere modification mediated by the Haspin kinase or other aspects of centromeric structure required for kinetochore assembly and imparting cohesion will be defective. Haspin mediates M phase-specific H3T3 phosphorylation (pH3T3) [28, 29], in a process guided and governed by the combinatorial action of mitotic kinases [30, 31]. We found that pH3T3 was strongly diffused in *ESCO2^−/W615G^ DDX11*^−/−^ cells, and to a lesser extent in the single *ESCO2^−/W615G^* and *DDX11*^−/−^ mutants (Figure 5A). pH3T3 occurs also along chromosome arms, but is concentrated at the inner centromere [28, 29]. The pathways underlying this distribution of pH3T3 and Haspin are multiple, and involve increased recruitment of Haspin at the centromere via Pds5 interaction with Haspin [32, 33] and via SUMOylated DNA topoisomerase II [34].

Because Pds5 is part of the cohesin complex, which is thought to become concentrated at centromeres after removal of cohesin from chromosome arms during prophase [27, 35], we suspected that the observed delocalization of pH3T3 at centromeres is due to a reduction of cohesin in the centromeric region. We thus addressed if the defect in pH3T3 concentration at the inner centromere in the analyzed mutants also correlates with reduced localization of cohesin in the centromeric regions (marked by CENP-T). However, we could not detect clear foci of cohesin at centromeres using staining techniques that either use or exclude triton for permeabilization (Supplementary Figure S5A) and could not chromatin immunoprecipate endogenously tagged cohesin subunits, Smc3 and Rad21 to centromeres (data not shown). Thus, our results do not support the conclusion that cohesin delocalization from centromeres underlies the observed pH3T3 diffusion, although, because of our failure to detect a cohesin pool at centromeres, we cannot rule out this possibility.

Because pH3T3 facilitates correct localization of Aurora B and Survivin [32, 36, 37], critical components of the chromosomal passenger complex (CPC) that are crucial for congression of chromosomes at the metaphase plate and for chromosome segregation [38], we tested Aurora B localization in our mutants. As expected, in similar trends with pH3T3 delocalization, we found Aurora B to be largely diffused to chromosome arms in *ESCO2^−/W615G^ DDX11*^−/−^ cells, while it was only mildly delocalized in *DDX11*^−/−^ and *ESCO2^−/W615G^* single mutants (Figure 5A).

Optimal CPC enrichment at centromeres involves besides pH3T3, H2AT120 phosphorylation by Bub1 kinase in the kinetochore-proximal region, which also promotes Shugoshin recruitment [39]. Differently from pH3T3, we found clear foci for KNL1, MAD2 and CENP-T (Supplementary Figure S5B), for phospho-Histone H2AT120 (pH2AT120) (data not shown), as well as for Shugoshin (Sgo1) (Supplementary Figure S5C). In all, the results indicate that *ESCO2* and *DDX11* mutations cause a defect primarily in the pH3T3 axis (Figure 5A).

To next measure the localization of pH3T3 on chromosomes in a quantitative manner, we performed ChIP analysis of pH3T3. In these experiments, we measured the ratio of pH3T3 immunoprecipitated at centromeres versus pH3T3 immunoprecipitated at a repetitive region present on chromosome arms (male hypermethylated (MHM)) region. We used the repetitive MHM region as reference, and not single copy DNA regions, in order to objectively assess that the enrichment of pH3T3 at centromeres is not simply due to increased amplification of repetitive DNA. Consistent with the immunofluorescence results, we found reduced pH3T3 concentration in both *DDX11*^−/−^ and *ESCO2^−/W615G^* cells at two tested centromere (CEN) regions, in comparison with WT (Figure 5B and data not shown). Moreover, further reduction was observed in *ESCO2^−/W615G^ DDX11*^−/−^ cells versus single mutants (Figure 5B). These results indicate that DDX11 and ESCO2 sustain the inner-centromere pH3T3 axis by separate mechanisms, and their cooperation is required for robust centromeric cohesion (Figure 3C-3D) and correct chromosome segregation (Figure 4).

### Tipin prevents degeneration of mild inner-centromere dysfunction of *ESCO2^−/W615G^* cells towards chromosome missegregation

The replication fork-protection Tim-Tipin complex is critical for replication fork elongation and promotes SCC [6, 40]. Previous work indicated that DDX11 and Tipin affect cohesion via a common pathway [6, 41], and Tim directly interacts with DDX11 [5]. Although to date *Tipin* mutations were not identified as driver alleles in cohesinopathy-like developmental disorders, we examined whether Tipin inactivation would resemble *DDX11*^−/−^ for the aspects analyzed above.

**Figure 6:**
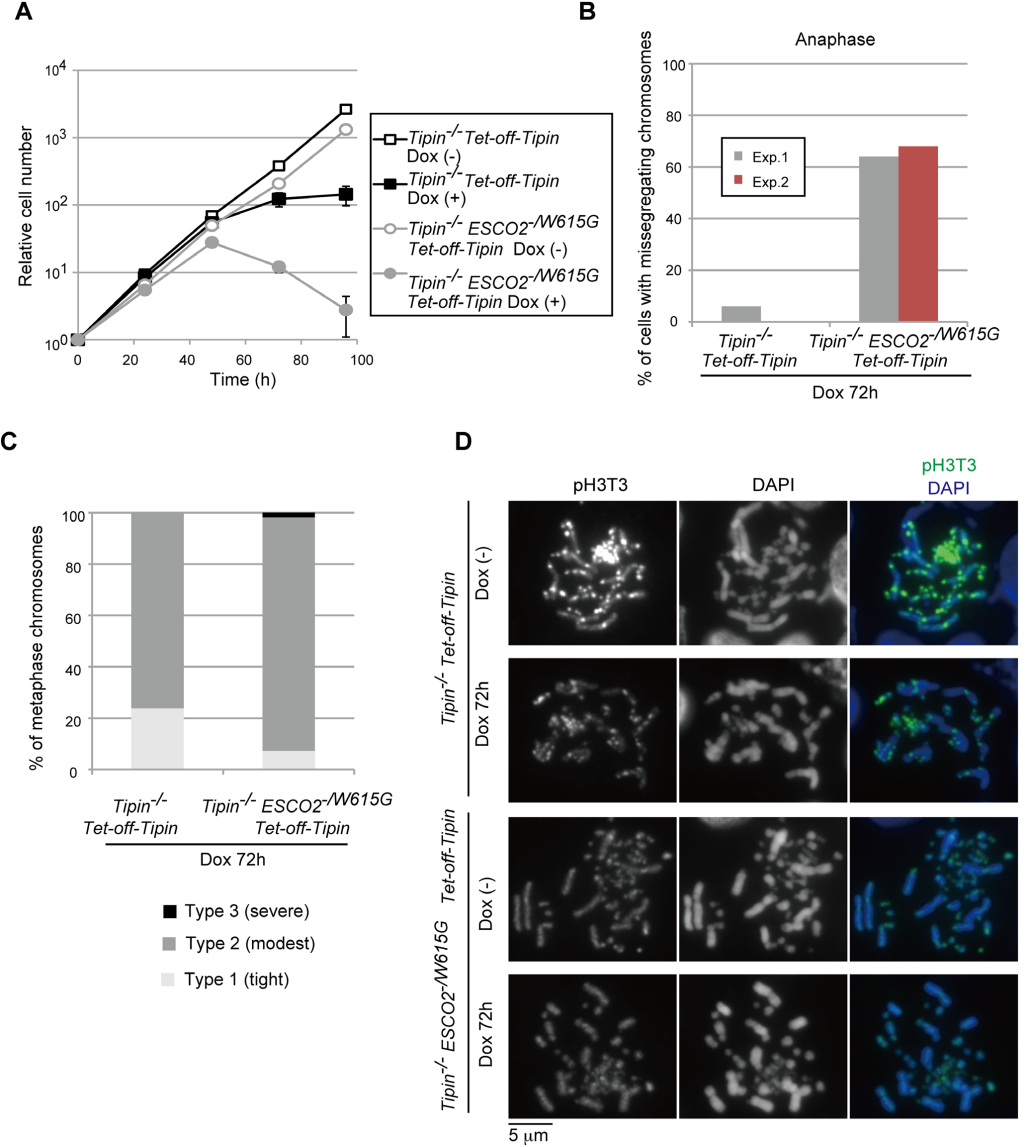
Tim-Tipin pathway is essential in *ESCO2^−/W615G^* cells **A.** Growth curves. **B.** Chromosomes were examined in the indicated genotypes as in Fig. 4D. 50 cells for each experiment were analyzed. The results of two independent experiments are plotted. **C.** Metaphase spreads, from cells incubated with Dox for 72 h, were classified for cohesion defects as in Figure 2B. More than 100 metaphase cells were examined. **D.** Localization of pH3T3 as in Figure 5A.

To these ends, we used *Tipin* conditional KO cells in which the exogenously expressed *cTipin* is suppressed by addition of Dox [40]. Next, we established conditional *Tipin^−/−^ ESCO2^−/W615G^* cell lines (Supplementary Figure S6A). *Tipin*^−/−^ cells showed only a modest drop in viability, whereas repression of *Tipin* expression for 48 hr in conditional *Tipin^−/−^ ESCO2^−/W615G^* cells halted proliferation and caused lethality (Figure 6A). Similarly to *ESCO2^−/W615G^ DDX11*^−/−^ cells, *Tipin^−/−^ ESCO2^−/W615G^* cells had high frequency of misaligned and missegregating chromosomes in (pro)metaphase and anaphase (Figure 6B and Supplementary Figure S6B-C).

Interestingly, differently from *DDX11^−/−^ ESCO2^−/W615G^,* the chromosome missegregation phenotype was neither preceded nor coincident with obvious premature centromere sister chromatid separation in *Tipin^−/−^ ESCO2^−/W615G^* metaphase cells, although Tipin depletion, similarly to *DDX11*^−/−^, associated with chromosome arm cohesion defects (Figure 6C). Thus, these results uncover mutants with very similar cohesion phenotypes in metaphase spreads (i.e. *Tipin*^−/−^ alone or *Tipin^−/−^ ESCO2^−/W615G^*), but very different behavior in mitosis (i.e. chromosome missegregation is much more severe in *Tipin^−/−^ ESCO2^−/W615G^* compared to *Tipin*^−/−^). Moreover, the results identify situations with high frequency of chromosome missegregation and in which centromeric separation is not visible (*Tipin^−/−^ ESCO2^−/W615G^*), or becomes visible much later than chromosome missegregation itself (*DDX11^−/−^ ESCO2^−/W615G^*).

Importantly, similarly with *DDX11^−/−^ ESCO2^−/W615G^* cells, the mitotic chromosome missegregation phenotype in *Tipin^−/−^ ESCO2^−/W615G^* again coincided with diffused pH3T3 (Figure 6D) and Aurora B (data not shown). We conclude that Tipin acts in a manner similar to DDX11, in preventing the inner-centromere dysfunction to degenerate further and cause chromosome missegregation. However, Tipin contribution to centromeric cohesion are likely much more subtle or fully compensated by DDX11. Because even mild cohesion defects such as those of *ESCO2^−/W615G^*, *Tipin*^−/−^ and *DDX11*^−/−^ single mutants, which did not cause any visible premature separation at the centromere (Figures 2B, 6D and 6C), led to diffused pH3T3 at the inner-centromere (Figures 5A, 6D), the results suggest that one of the first consequences of reduced cohesion is a dysfunction of the inner-centromere, which can then further degenerate towards chromosome missegregation and/or centromeric cohesion defects.

## DISCUSSION

Our work reveals that the evolutionarily conserved Tim-Tipin fork protection complex, DDX11 helicase and ESCO2 acetyltransferase collaborate in several respects relevant for chromosome structure and genome integrity. In the context of chromatid cohesion, our findings indicate that DDX11 and ESCO2 are critical for centromeric cohesion, in which context they play partially overlapping roles, being able to compensate for each other to prevent overt centromeric separation (Figure 3B-3E).

Our results show that DDX11 is critical for maintenance of cohesion on chromosome arms even in the presence of ESCO2 (Figure 2B). This function of DDX11, involving its helicase activity (Figure 2B), is likely conducted jointly with its interacting partner, Tim-Tipin, mutations in which resemble *DDX11*^−/−^ (Figures 2B, 3B-3C, 4C-4D, 6A-6C) [5, 6]. Interestingly, however, we observed lethality between *ESCO2^−/W615G^* and *DDX11*^−/−^ in the presence of Tipin, and between *ESCO2^−/W615G^* and *Tipin*^−/−^ in the presence of DDX11. These results indicate that Tipin and DDX11 functions are not redundant, but perhaps act in compensatory fashion with respect to managing chromosome-related processes that impact on cohesion. That DDX11 and Tipin functions will be different and non-redundant is also evidenced by our observation that DDX11 has a much stronger contribution than Tipin in providing for centromeric cohesion in *ESCO2^−/W615G^* cells (Figures 3C and 6C), although both *ESCO2^−/W615G^ Tipin*^−/−^ and *ESCO2^−/W615G^ DDX11*^−/−^ conditional cells are dying (Figures 3B and 6A). The most likely cause of death in both *ESCO2^−/W615G^ Tipin*^−/−^ and *ESCO2^−/W615G^ DDX11*^−/−^ cells is related to rampant chromosome missegregation events (Figures 4D and 6B), which are explained by the strong delocalization of pH3T3 and Aurora B from the centromeres in these mutants (Figures 5 and 6D). In all, these results reveal that the inner-centromere function involving pH3T3 accumulation is enabled collectively by DDX11, Tipin and ESCO2. Subtle inner-centromere dysfunction is then further prevented from leading to overt centromeric separation by DDX11 and ESCO2 (see Figures 3C and 4D). These results suggest that robust centromeric cohesion and inner-centromere functions are enabled and protected by overlapping mechanisms, but with partly different kinetics.

The observed cohesion and inner-centromere defects could be, in principle, the simple consequence of prolonged mitosis. However, we found that also in single *DDX11*^−/−^ and *ESCO2^−/W615G^* mutants that proliferate normally, pH3T3 was delocalized (Figures 2 and 5). DDX11, Tipin and ESCO2 link replication with cohesion [10, 15], and promote replication fork progression, especially in difficult-to-replicate regions [5, 42]. Notably, replication stress, such as fork topology and fork speed alterations that were reported in DDX11, Tipin and ESCO2 mutants [5, 42, 43], can associate with cohesion defects [15, 16, 43]. Thus, while it is formally possible that DDX11, Tipin and ESCO2 have a more direct role in cohesion regulation independent of replication, we favor the idea that replication problems arising in their absence play an important role in the etiology of the cohesion defects and pH3T3 delocalization observed in the corresponding mutants.

How are the cohesion and pH3T3 defects linked to each other? Centromeric cohesion is maintained both via cohesin and topological-links or catenations, and both these factors influence the recruitment of Haspin to the centromere [32-34], which stabilizes cohesin [44]. However, if the role of Haspin in cohesion involves its kinase activity or pH3T3 is not known. Because in all analyzed cohesion mutants we observed diffused pH3T3 accumulation, regardless of whether sister chromatid separation can be observed by classically employed cytogenetic approaches, we propose that pH3T3 is a very sensitive marker of cohesion insufficiency. Moreover, because mild pH3T3 delocalization can be exacerbated to cause chromosome missegregation, associated or not with overt centromeric separation, our findings indicate that pH3T3 act to enhance cohesion, conducting to robust centromeric cohesion with the help of ESCO2 and DDX11.

How may replication stress negatively influence cohesion and pH3T3? While more work is clearly needed to answer this question, we speculate that complex topological structures and abnormal replication intermediates shown to accumulate at centromeres in cells defective in Tipin [43, 45], and likely also in DDX11 mutants [5], ultimately perturb the establishment of a proper chromatin environment involving pH3T3. This would not be unprecedented as perturbations in the replication of other complex genomic structures can affect histone modifications [46, 47]. Importantly, this concept opens new lines of research for investigation the role of replication factors in cohesion and genome integrity, namely, by their roles in engendering a chromatin environment enriched in pH3T3.

In conclusion, we uncovered specific molecular defects appearing in distinct cohesinopathy-like conditions that affect the faithfulness of chromosome segregation, with or without a marked impact on cohesion or the proliferation speed. Our work reveals that mild or even invisible cohesion defects could be exacerbated by additional mutations to reach lethality by further impairing the inner-centromere dysfunction. An implication of these findings is that in cancers with mutations in cohesion factors or experiencing certain forms of replication stress, inner-centromere dysfunction and cohesion defects could be specifically exacerbated to produce selective killing of those cells. We propose that suboptimal pH3T3 concentration at the inner-centromere is a useful indicator of cohesion insufficiency and one important chromatin determinant of postreplicative cohesion.

## MATERIALS AND METHODS

The cell lines are listed in Table S1, and the knock-out and knock-in constructs are described in the Supplementary Information. mRNA isolation, reverse transcription PCR, Western blotting and cell cycle analysis were performed as described [48]. Live cell imaging, immunofluorescence analysis, and ChIP-qPCR followed reported procedures and are described in the Supplementary Information.

## SUPPLEMENTARY MATERIALS FIGURES AND TABLE


