# Supplementary material for: Chromatin determinants of the inner-centromere rely on replication factors with functions that impart cohesion
Source: Oncotarget. 2016 Sep 12;7(42):67934–47. doi: 10.18632/oncotarget.11982 (PMC5356530; doi:10.18632/oncotarget.11982)
Supplement: Supplementary file 1 [file oncotarget-07-67934-s001.pdf]

Chromatin determinants of the inner-centromere rely on replication factors with functions that impart cohesion

Supplementary Material

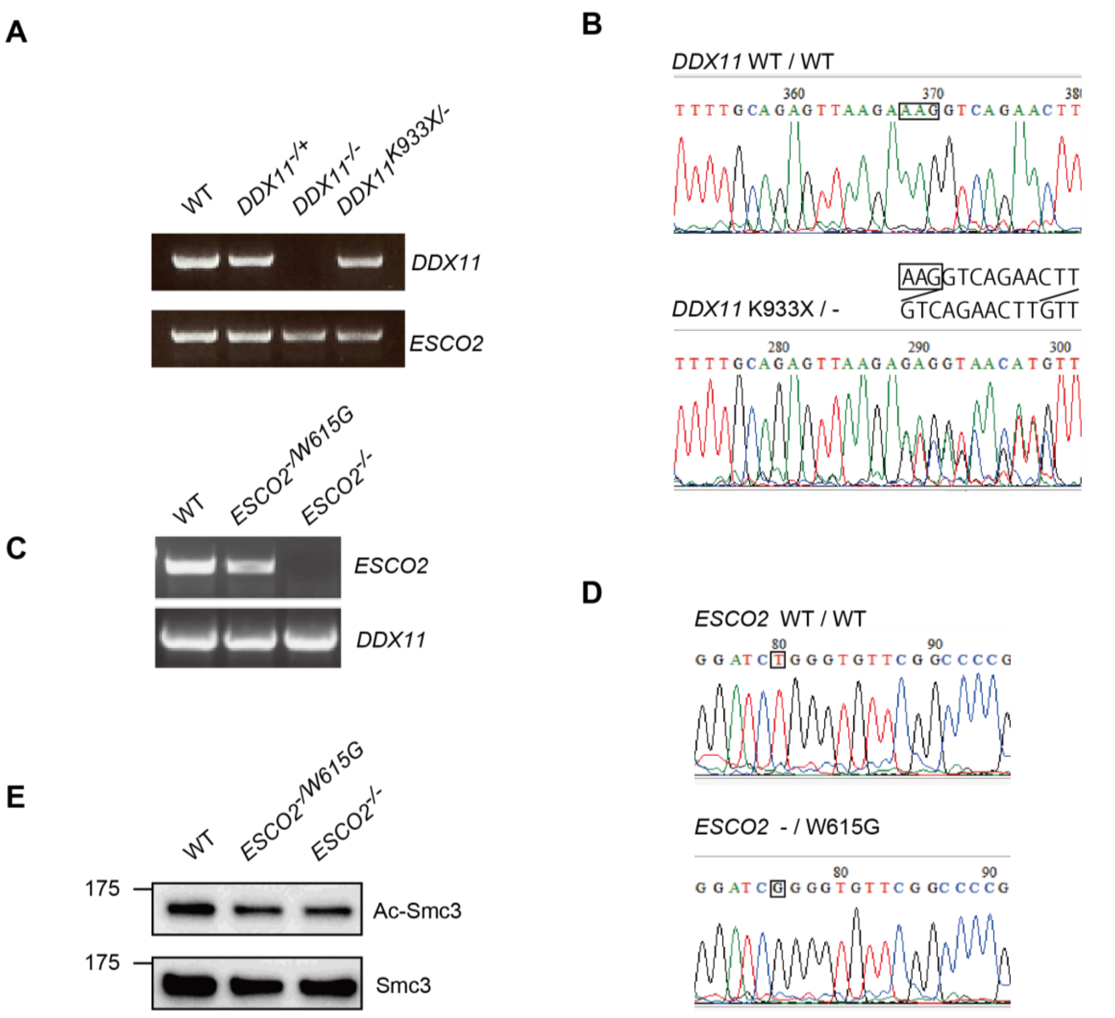

Supplementary Figure S1. Validation of *DDX11* and ESCO2 mutations and their effect on Smc3 acetylation. (A) Confirmation of *DDX11* gene knockout and presence of *ESCO2* mRNA expression by RT-PCR in the indicated cell lines. The results were confirmed from a different biological replicate experiment. (B) DNA sequencing of the genomic DNA *DDX11* locus in WT and *DDX11*$^{K933X/-}$ cells. AAG sequence (enclosed by a square) was deleted from one allele of *DDX11* gene, and the other DDX11 allele was replaced by a *DDX11* knockout construct (Figure 1A), in which however the genomic portion corresponding to K933 remains intact. (C) Confirmation of the presence of

ESCO2 and *DDX11* mRNA expression by RT-PCR in the indicated cell lines. The results were confirmed from a different biological replicate experiment. (D) DNA sequencing of *ESCO2* locus in *ESCO2*$^{-/W615G}$ cells. One allele of this locus was knocked out and the other allele has a T to G mutation (enclosed by a square) at the *ESCO2* gene. The engineered mutation causes an aminoacid change, from Trp (W, encoded by UGG) to Glycine (GGG). (E) Total cell lysates were prepared from cells of the indicated genotypes and analyzed by Western blotting for Ac-Smc3 and total Smc3. Western blots were performed at least twice from independent biological replicates and one representative result is shown.

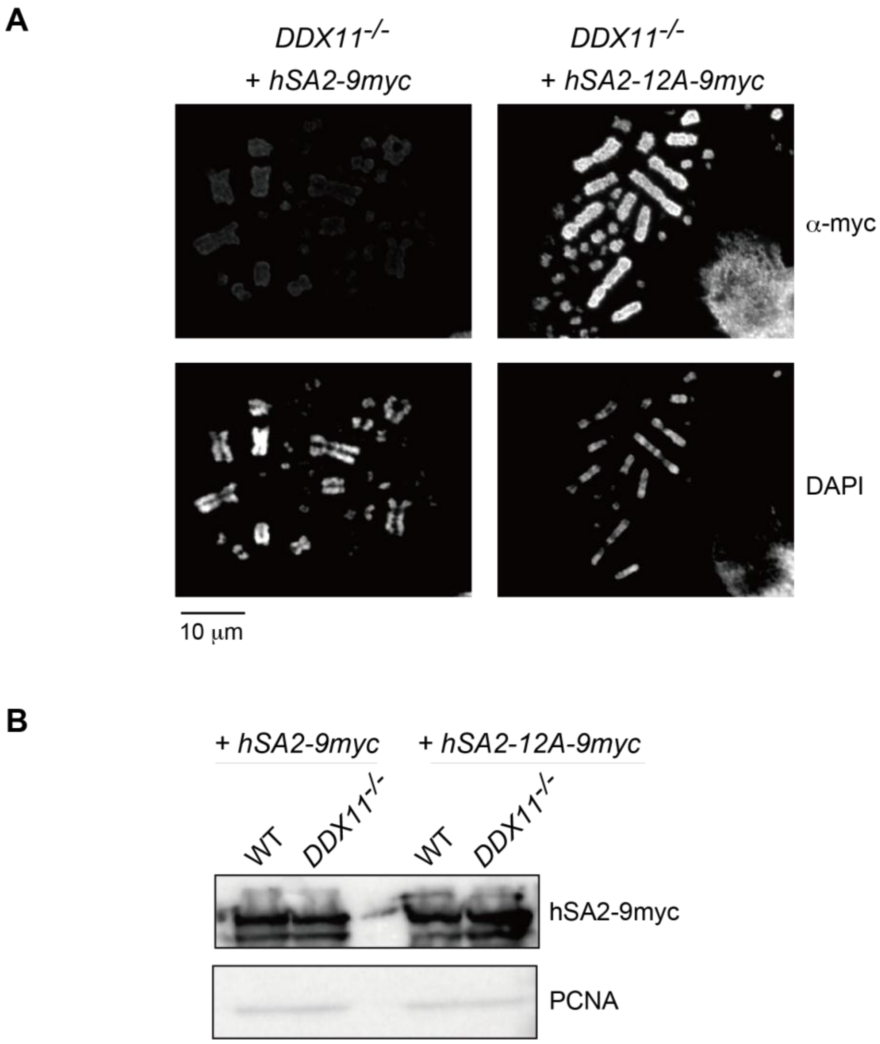

Supplementary Figure S2. Chromatin association and expression levels of hSA2-9myc or hSA2-12A-9myc in *DDX11*$^{-/-}$ cells. (A) Cells were incubated with colcemid for 1 h and metaphase spread samples were prepared by the cytospin method. Chromosomes were visualized by DAPI staining. The same trend was confirmed using an independent biological experiment. (B) Expression levels of hSA2 and hSA2-9Myc in WT and *DDX11*$^{-/-}$ cells.

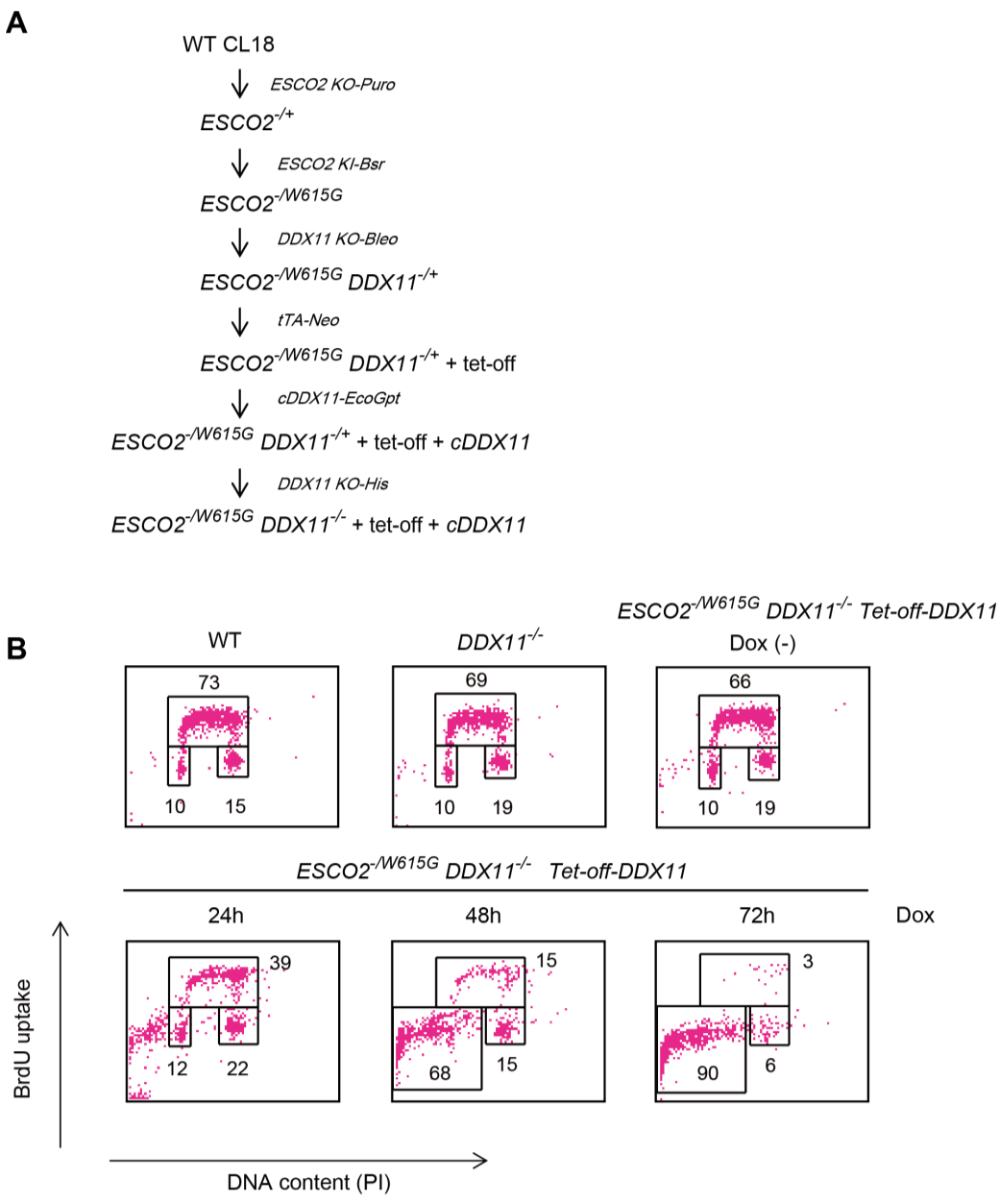

Supplementary Figure S3. Establishment of *ESCO2*$^{-/W615G}$ *DDX11*$^{-/-}$ cell lines and their cell-cycle characterization. (A) Schematic representation of the generation of conditional *ESCO2*$^{-/W615G}$ *DDX11*$^{-/-}$ mutant. (B) Cell cycle distribution of cells of the indicated genotype. Cells were cultured in the presence of Dox for the indicated times, pulse-labeled with BrdU for 15 min, and harvested. The cells were stained with FITC anti-BrdU to detect BrdU uptake and with propidium iodide (PI) to detect DNA. The vertical axis represents BrdU uptake and horizontal axis represents total DNA. The gates represent G1 (or G1 + SubG1 (apoptotic cells)), S and G2/M phase. The numbers show the percentage of cells in each gate. The results were confirmed with an independent biological replicate experiment.

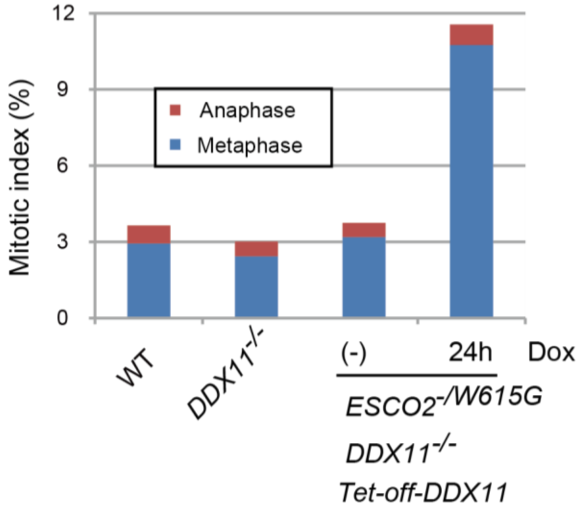

Supplementary Figure S4. Mitotic index in WT, *DDX11*$^{-/-}$, *ESCO2*$^{-/W615G}$, and *ESCO2*$^{-/W615G}$ *DDX11*$^{-/-}$ cell lines. Asynchronous cells spun onto slide glasses by cytospin method. Nuclei were visualized by DAPI staining. Approximately 1000 cells were counted and populations of metaphase and anaphase cells were plotted. The results were confirmed with an independent biological replicate experiment.

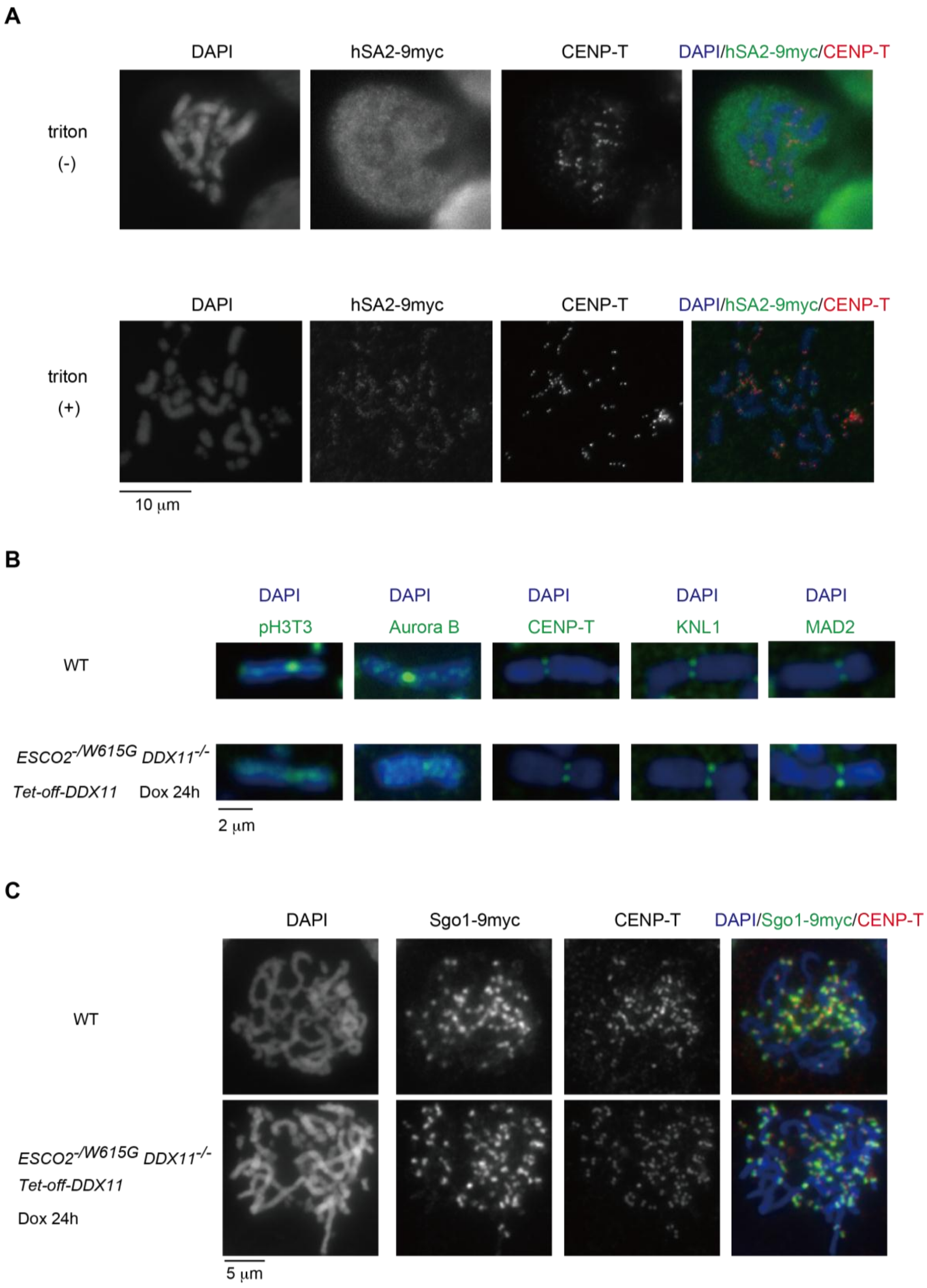

Supplementary Figure S5. Inner-centromere structure, but not localization of kinetochore proteins is affected in *ESCO2*$^{-/W615G}$ *DDX11*$^{-/-}$. (A) Localization of cohesin (SA2-9Myc) versus CENP-T in WT cells in metaphase cells using two

types of staining conditions. Specific foci of cohesin and co-localization with CENP-T was not observed in either condition. (B) WT and *ESCO2*$^{-/W615G}$ *DDX11*$^{-/-}$ cells were stained as described in Figure 5A for pH3T3, Aurora B, CENP-T, MAD2 and KNL1. An enlargement of the foci formed on chromosomes is shown. (C) Localization of Sgo1-9myc in WT and *ESCO2*$^{-/W615G}$ *DDX11*$^{-/-}$ prometaphase cells in the presence of Dox. (A-B)Cells were incubated with colcemid for 1 h and metaphase spread samples were prepared by cytospin method. Chromosomes were visualized by DAPI staining. For A-C, the same trends were confirmed from an independent biological replicate experiment.

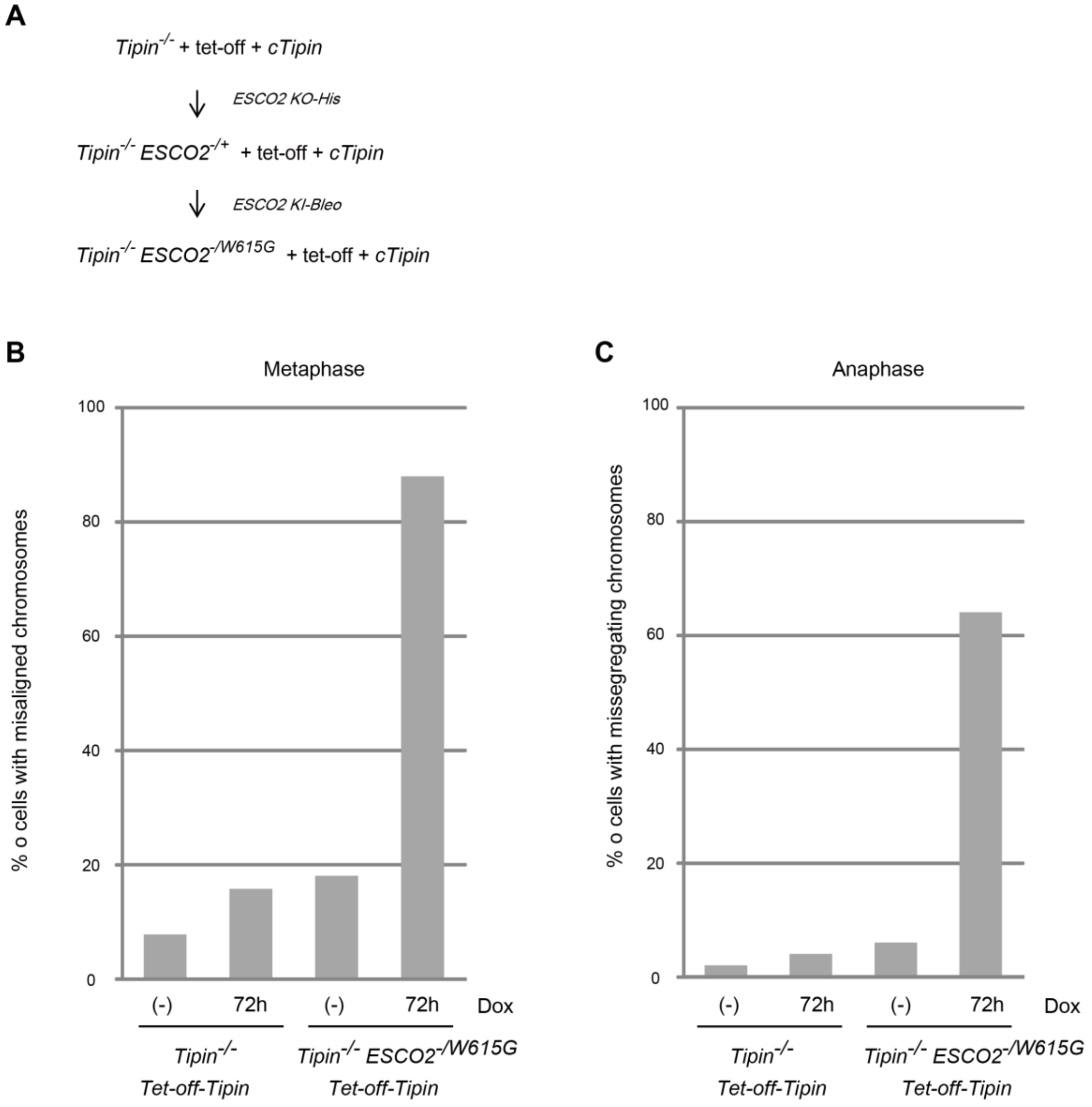

Supplementary Figure S6. Establishment and characterization of *Tipin*$^{-/-}$ *ESCO2*$^{-/W615G}$ mutants. (A) Schematic representation of how conditional *Tipin*$^{-/-}$ *ESCO2*$^{-/W615G}$ mutants were generated. (B-C) The percentage of cells with misaligned chromosomes in metaphase (B) or anaphase (C) in the indicated cell lines and conditions. Over 100 cells for the metaphase plots and 50 cells for the anaphase plot were analyzed.

Table S1. Cell lines used in this study.

Genotype	Selective marker	Reference
DT40 WT CL18		[1]
DDX11$^{-/-}$	*DDX11/DDX11*::KO-Puro/KO-Bsr	This study
DDX11K933X/-	*DDX11/DDX11*::KI-Bsr/KO-Puro	This study
DDX11$^{-/-}$ + *GFP-cDDX11-HA*	*DDX11/DDX11*::KO-Puro/KO-Bsr, +GFP-c*DDX11*-HA::Neo	This study
DDX11$^{-/-}$ + *GFP-cDDX11*(K87A)*-HA*	*DDX11/DDX11*::KO-Puro/KO-Bsr, +GFP-cDDX11-HA::Neo	This study
DDX11$^{-/-}$ + *hSA2-9myc* (tet-on)	*DDX11/DDX11*::KO-Puro/KO-Bsr, +tet3G::Neo, +hSA2-9myc::His	This study
DDX11$^{-/-}$ + *hSA2-12A-9myc* (tet-on)	*DDX11/DDX11*::KO-Puro/KO-Bsr, +tet3G::Neo, *+hSA2-12A*-9myc::His	This study
ESCO2$^{-/W615G}$	*ESCO2/ESCO2*::KO-Bsr/KI-Puro	This study
ESCO2$^{-/W615G}$ *DDX11*$^{-/-}$ + *cDDX11-HA* (tet-off)	*ESCO2/ESCO2*::KO-Bsr/KI-Puro, *DDX11/DDX11*::KO-Bleo/KO-His, +tet off::Neo, +c*DDX11*-HA::Eco	This study
Tipin$^{-/-}$ + *cTipin-FLAG* (tet-off)	*Tipin/Tipin*::KO-Bsr/KO-Puro, tet off c*Tipin*-FLAG::Hyg	[2]
Tipin$^{-/-}$ *ESCO2*$^{-/W615G}$ + *cTipin-FLAG* (tet-off)	*Tipin/Tipin*::KO-Bsr/KO-Puro, tet off c*Tipin*-FLAG::Hyg, *ESCO2/ESCO2*::KO-His/KI-Bleo	This study
DT40 Cre1	+Cre::Neo, +V-myb::His	[3]
ESCO2$^{-/-}$	+Cre::Neo, +V-myb::His, *ESCO2/ESCO2*::KO-Puro /cKO-loxP-Bsr-LoxP (removed)	This study
WT + c*Sgo1-9myc*	+cSgo1-9myc :: His	This study
ESCO2$^{-/W615G}$ *DDX11*$^{-/-}$ + *cDDX11* (tet-off) + *Sgo1-9myc*	*ESCO2/ESCO2*::KO-Bsr/KI-Puro, *DDX11/DDX11*::KO-Bleo/KO-Hyg, +tet off::Neo, +c*DDX11*-HA::Eco, +cSgo1-9myc :: His	This study

Experimental Procedures

Plasmid construction and transfection

DDX11 KO-Bsr and DDX11 KO-Puro were generated from genomic PCR products combined with Blasticidin S and Puromycin selection marker cassettes. Genomic DNA sequences were amplified using the primers 5’-GAGCAGCTGGATCACAACGAGGAGGAGCTG-3’ and 5’-CACATACCTGCCTGGATCCCAAGGAGACC-3’ (for the left arm of the KO construct); and 5’-GGATCCGAAGTGAGGAATACAGCAGGTGTGAC-3’ and 5’-ATGCACAAGCGGAGGATGTCTGTACTCACC-3’ (for the right arm of the KO construct), Amplified PCR products were cloned into pGEM-T Easy vector (Promega). The 1.9 kb SalI (in pGEM-T easy)-BamHI (in genomic DNA) fragment from the left arm was cloned into SalI/BamHI sites of right arm (2.2 kb) sub-cloned pGEM-T easy vector. The BamHI site was next used to clone marker gene cassettes. In the same strategy, left arm and right arm of ESCO2 KO-Puro were amplified using the primers 5’-GTCCACCTGCAAAAATGCCTCACGGTGTG-3’ and 5’-GGATCCGGCATCCTAGTGAGAAACAGAGCTGG-3’ (for the left arm of the KO construct); and 5’-GGATCCAAACACCCACCTTCCTCGTCTACAAC-3’ and 5’-AAGGGTGAAGCAGTTGATTTGGGCACTTCC-3’ (for the right arm of the KO construct). The 1.5 kb NdeI (in pGEM-T easy)-BamHI (attached by PCR) fragment from the left arm was cloned into NdeI/BamHI sites of right arm (2.1 kb) cloned pGEM-T easy vector. To make DDX11 KI-Puro, 4.3 kb fragment was amplified using the primers 5’-GTAATTCATCCTTCCTTGGACAGACTGGGG-3’ and 5’-CAGTGGAGCCTTCACAAGCAGGTATTTCC-3’ and cloned into pGEM-T easy vector. Using this as a template, we amplified an AAG deleted fragment (which corresponds to K933 of DDX11) using the primers 5’-GTCAGAACTTGTTTCTCACATACTGCTGCAG-3’ and 5’-TCTTAACTCTGCAAAAGCTGATCCAAAGGCAGG-3’. Puromycin selection marker cassette was inserted at the BglII site of this vector. To make ESCO2 KI-Bsr, 3.5 kb fragment was amplified using the primers 5’-GTGCTGTCTGAGCCCAGCACAGTGCACAG-3’ and 5’-AAGGGTGAAGCAGTTGATTTGGGCACTTCC-3’ and cloned into pGEM-T easy vector. Using this as a template, we substituted a T to G using the

primers 5’-GGGGTGTTCGGCCCCGCGC-3’ and 5’-GATCCTGCTGACACCACACACTGCAGGC-3’ to introduce the W615G mutation. BamHI site was introduced to the vector using the primers 5’-GGATCCCACCACCACCCCAAGGACAC-3’ and 5’-ACCCCTCAAGCTAATAGTAGGGCCAC-3’. The puromycin selection marker cassette was inserted at this BamHI site of this vector. Chicken DDX11 cDNA was amplified using the primers 5’-ATGGCGGGACGGCGGAGGTGGGCGGCGGGCTGGCGGCTGGGAGCTGACAG-3’ and 5’-AGCTTCTAGATTAAGCGTAGTCTGGGACGTCGTATGGGTAGTCTGATTTCCCTCGGTGGAAC-3’ and cloned into the pGEM-T easy vector. *DDX11-HA* cDNA was then cloned into pEGFPC1 vector (clontech) or pUHG 10-3. DDX11-K87A mutant was made by site-directed mutagenesis using the primers 5’-GGGCACGGGAAGGTCGCTGAGCCTCATCTG-3’ and 5’-CAGATGAGGCTCAGCGACCTTCCCGTGCCC-3’. Amplified PCR products were cloned into pLoxP vectors [4]. These vectors were transfected to DT40 cells as previously described [5].

To generate the ESCO2 conditional KO-Bsr construct, three DNA fragments were amplified using the primers 5’-ATCGCGGCCGCTCTGTGCAGGGTCACCAACCAGCAG-3’ (*Not*I) and 5’-GCAACTAGTATTCTCATCTCTGTACCTCCCTCAGAG-3’ (*Spe*I), 5’-GAGAATGCTAGCAAGAGCCATAGAATC-3’ (*Nhe*I) and 5’-AGTGCTAGCCCCTCAAGCTAATAGTAGGGCCAC-3’ (*Nhe*I), 5’-ACAATCGATGTTTCCCACCACCACCCCAAGGAC-3’ (*Cla*I) and 5’-AGCGGTACCTCTGCAGCATCAGGTGCTGAGCAG-3’ (*Kpn*I), respectively and subsequently cloned into pLoxP-Bsr vector [3] using attached restriction sites (Supplementary Figure 2A).

To establish the Sgo1-9myc construct, chicken Sgo1 cDNA was amplified using the primers 5’-GCTAGCTCGAAGATGGCTGAGCACCTGAAAAAGC-3’ (*Nhe*I) and 5’-AGTGCGGCCGCAACAACCACCAACAAATTTCTCG-3’ (*Not*I) and then cloned into pTRE2-hygro vector (Clontech) with 9xmyc tag fused in frame [6]. The promoter was replaced by chicken beta-actin promoter and the hygromycin-resistant marker was replaced by the histidinol-resistant marker.

Cell culture

Cells were cultured at 39.5°C in D-MEM/F-12 medium (Gibco) supplemented with 10% fetal bovine serum, 2% chicken serum (Sigma), Penicillin/Streptomycin mix, and 10 μM 2-mercaptoethanol (Gibco) in the presence or absence of 1 μg/ml Dox. 30 μg of linearized vectors were transfected to 10^7 cells to obtain cell lines as indicated. The histone H2B-mCherry plasmid was transiently expressed by neon electroporation. To plot growth curves, each cell line was cultured in three different wells of a 24 well-plate and passaged every 12 h or 24 h. Cell number was determined by flow cytometry using plastic microbeads (07313-5; Polysciences). Cell solutions were mixed with the plastic microbead suspension at a rate of 10:1, and viable cells determined by forward scatter and side scatter.

Western blotting

Western blotting was performed as previously described [7] using antibodies against α-tubulin (Sigma, T5168), Ac-Smc3 [8], Smc3 [9], HA (Roche, 11 867 423 001) followed by horseradish peroxidase-conjugated anti-rabbit, anti-rat, or anti-mouse IgG secondary antibody (Cell Signaling). Proteins were visualized using SuperSignal West Femto Maximum Sensitivity Substrate (Thermo scientific).

Cell cycle analysis by flow cytometry

Flow cytometry was performed as previously described [5] [10]. Cells were cultured in the presence of BrdU for 15 min, fixed in 70% ethanol, incubated with anti-BrdU antibody (BD Biosciences, 555627), and stained with FITC-conjugated anti-mouse IgG antibody (Jackson ImmunoResearch, 115-096-072) and propidium iodide. For PI single staining analysis, cells were fixed in 70% ethanol and stained with propidium iodide.

mRNA isolation, reverse transcription PCR

Total RNA was isolated using TRIzol reagent (Invitrogen) and converted to cDNA with Superscript III (Invitrogen) as previously described [11]. The primers used in RT-PCR to amplify DDX11 and ESCO2 cDNA were 5'-

ATGGCGGGACGGCGGAGGTG-3' and 5'-GAGTTGACCGACAGCCCGCTTCTCTG-3', and 5'-ATGAGAAGCGGATGGGCGGCCTG-3', and 5'-CAGTGCACATCAAATGCCCTCCACCCAACC-3', respectively.

Living cell observation

For live cell imaging, a histone H2B-mCherry plasmid, purchased from Addgene and described in [12] was transfected into WT and mutant cells to visualize chromosomes as previously described [13]. Fluorescently stained living cells were observed with delta Vision microscope with an oil immersion objective lens in a temperature-controlled box to keep the temperature at 39.5°C. Time-lapse images were recorded at 3 min intervals. Subsequent analysis and processing of images were performed using ImageJ (NIH).

Immunofluorescent visualization of metaphase cells and metaphase spread sample

Immunocytochemical analysis was performed as described in [14]. Cells were spun onto slides with a cytocentrifuge and fixed by 4% paraformaldehyde in PBS for 10 min at room temperature. For metaphase spread samples, cells were treated for 1 h with medium containing 0.1 microgram/ml colcemid (GIBCO-BRL, Grand Island, NY). Harvested cells were treated in 500 μl of 0.075 M KCl for 10 min at room temperature and spun onto slides with a cytocentrifuge and fixed by 4% paraformaldehyde in PBS for 10 min at room temperature. Fixed cells were then permeabilized by 0.5% triton in PBS for 10 min at room temperature, rinsed by 0.5% BSA, and incubated for 1 h at room temperature with primary antibody. Binding of primary antibody was then detected with Cy3- or Alexa488-conjugated goat anti–rabbit IgG or goat anti-mouse IgG (molecular probes) diluted to an appropriate concentration in PBS/0.5% BSA. Affinity-purified rabbit polyclonal antibodies were used against recombinant chicken CENP-T [15], pH3T3 (Millipore, 07-424), MAD2, KNL1 and Aurora B [16]. The Myc antibody (home-made 9E10 clone) was used to detect 9myc-tagged proteins. Chromosomes and nuclei were counterstained with DAPI at 0.2 μg/ml. Immunofluorescence images were

collected with a 100× NA 1.40 objective lens together with a filter wheel at room temperature. Subsequent analysis and processing of images were performed using Meta-Morph software (Roper Scientific) and Image J (NIH). All images were scaled and processed identically.

ChIp-qPCR

Chromatin immunoprecipitation was performed as described in [17] with small modifications. 1×10^8 DT40 cells of the indicated genotype were synchronized by 200 ng/ml of Nocodazole and crosslinked with 1% formaldehyde (Electron Microscopy Sciences) in 8 ml of media for 5 min at room temperature, and incubated in media containing 200 mM glycine for 5 min to quench reactive aldehydes. Cells were immersed in 1 ml lysis buffer (10 mM Tris-HCl [pH 8.0], 10 mM NaCl, and 0.5% NP-40) for 5 min. Cells were collected by centrifugation (1500×g; 5 min; 4°C), washed once with ice-cold PBS, and resuspended in 1 ml Buffer A (15 mM Hepes-NaOH pH 7.4, 15 mM NaCl, 60 mM KCl, 0.34 M sucrose, 0.5 mM Spermidine, 0.15 mM Spermine, 1 mM DTT, 1mM $CaCl_2$ and protease inhibitors). Cells were pre-heated at 37°C for 5 min and treated with 3 μl micrococcal nuclease (Takara; 20 U/μl stock). The reaction tube was then incubated at 37°C for 1 h. After adding 20 μl 0.5 M EDTA [pH 8.0] (10 mM) and centrifugation (18,000×g; 5 min; 4°C), the pellet was suspended in 400 μl SDS lysis buffer (50 mM Tris-HCl [pH 8.0], 10 mM EDTA, and 0.5% SDS) and diluted by 600 μl ChIP dilution buffer (50 mM Tris-HCl [pH 8.0], 167 mM NaCl, 1.1% Triton X-100, 0.055% sodium deoxycholate, and protease inhibitor) before sonication (Branson Sonifier 250 with microtip; 5 times for 10 sec). After centrifugation to remove insoluble materials, 3 ml ChIP dilution buffer was added to the supernatant to yield the input for ChIP.

For cross-linked ChIP, Dynabeads (Dynabeads® Protein A; Invitrogen) were washed with PBS, incubated with Phospho-histone H3T3 (pH3T3) antibody (Millipore) or normal rabbit IgG, in 500 μl RIPA-150 mM NaCl over 3 h at 4°C with rotation and washed twice with 1 ml RIPA-150 mM NaCl. ChIP input (4 ml) was incubated with antibody-bound Dynabeads for 2 h at 4°C with rotation. Beads were washed with 1 ml RIPA-150 mM NaCl 4 times. Beads were mixed with 200 μl direct elution buffer (10 mM Tris-HCl [pH 8.0], 300 mM

NaCl, 5 mM EDTA, and 0.5% SDS) and incubated overnight at 65°C to reverse cross-linking. Samples were then treated with RNase A (Sigma; 5 μg/ml; 37°C; 30 min) and proteinase K (Roche; 200 μg/ml; 55°C; 3 h). DNA was extracted sequentially with phenol-chloroform (1:1) and precipitated with ethanol. After centrifugation, the pellet was washed with 70% ethanol and finally dissolved in 40 μl TE. Each sample (1 μl equivalent) was analyzed by real-time PCR (Promega; GoTaq® qPCR Master Mix) using LightCycler® 480 (Roche). The ratios of pH3T3 level between Cen2 and MHM repeats (Arm) were normalized by those of Input samples. We chose MHM locus as control due to its repetitive nature. With this control we could obtain reproducible results even from very low amount of DNA. Primer sequences were: Cen2 F, 5'- TGTGCAGACAAGCTCACCCTTATC-3'; Cen2 R, 5'-AAAGCAACACCCCTTCTTTCTGCG-3'; MHM F, 5'-CATTTTGTTGGAGCCTGCGTTCCAC-3'; MHM R, 5'-GCCTGGGAATATGAAGGAGCTAAAG-3'

References

1. Buerstedde JM and Takeda S. Increased ratio of targeted to random integration after transfection of chicken B cell lines. Cell. 1991; 67(1):179-188.
2. Hosono Y, Abe T, Higuchi M, Kajii K, Sakuraba S, Tada S, Enomoto T and Seki M. Tipin functions in the protection against topoisomerase I inhibitor. J Biol Chem. 2014; 289(16):11374-11384.
3. Arakawa H, Hauschild J and Buerstedde JM. Requirement of the activation-induced deaminase (AID) gene for immunoglobulin gene conversion. Science. 2002; 295(5558):1301-1306.
4. Arakawa H, Lodygin D and Buerstedde JM. Mutant loxP vectors for selectable marker recycle and conditional knock-outs. BMC Biotech. 2001; 1:7.
5. Sonoda E, Sasaki MS, Buerstedde JM, Bezzubova O, Shinohara A, Ogawa H, Takata M, Yamaguchi-Iwai Y and Takeda S. Rad51-deficient vertebrate cells accumulate chromosomal breaks prior to cell death. EMBO J. 1998; 17(2):598-608.
6. Hauf S, Roitinger E, Koch B, Dittrich CM, Mechtler K and Peters JM. Dissociation of cohesin from chromosome arms and loss of arm cohesion

during early mitosis depends on phosphorylation of SA2. PLoS Biol. 2005; 3(3):e69.

7. Abe T, Ishiai M, Hosono Y, Yoshimura A, Tada S, Adachi N, Koyama H, Takata M, Takeda S, Enomoto T and Seki M. KU70/80, DNA-PKcs, and Artemis are essential for the rapid induction of apoptosis after massive DSB formation. Cell Signal. 2008; 20(11):1978-1985.

8. Deardorff MA, Bando M, Nakato R, Watrin E, Itoh T, Minamino M, Saitoh K, Komata M, Katou Y, Clark D, Cole KE, De Baere E, Decroos C, Di Donato N, Ernst S, Francey LJ, et al. HDAC8 mutations in Cornelia de Lange syndrome affect the cohesin acetylation cycle. Nature. 2012; 489(7415):313-317.

9. Losada A, Hirano M and Hirano T. Identification of Xenopus SMC protein complexes required for sister chromatid cohesion. Genes Dev. 1998; 12(13):1986-1997.

10. Era S, Abe T, Arakawa H, Kobayashi S, Szakal B, Yoshikawa Y, Motegi A, Takeda S and Branzei D. The SUMO protease SENP1 is required for cohesion maintenance and mitotic arrest following spindle poison treatment. Biochem Biophys Res Commun. 2012; 426(3):310-316.

11. Abe T and Branzei D. High levels of BRC4 induced by a Tet-On 3G system suppress DNA repair and impair cell proliferation in vertebrate cells. DNA Repair. 2014; 22:153-164.

12. Nam HS and Benezra R. High levels of Id1 expression define B1 type adult neural stem cells. Cell Stem Cell. 2009; 5(5):515-526.

13. Amano M, Suzuki A, Hori T, Backer C, Okawa K, Cheeseman IM and Fukagawa T. The CENP-S complex is essential for the stable assembly of outer kinetochore structure. J Cell Biol. 2009; 186(2):173-182.

14. Hori T, Shang WH, Toyoda A, Misu S, Monma N, Ikeo K, Molina O, Vargiu G, Fujiyama A, Kimura H, Earnshaw WC and Fukagawa T. Histone H4 Lys 20 monomethylation of the CENP-A nucleosome is essential for kinetochore assembly. Dev Cell. 2014; 29(6):740-749.

15. Hori T, Amano M, Suzuki A, Backer CB, Welburn JP, Dong Y, McEwen BF, Shang WH, Suzuki E, Okawa K, Cheeseman IM and Fukagawa T. CCAN makes multiple contacts with centromeric DNA to provide distinct pathways to the outer kinetochore. Cell. 2008; 135(6):1039-1052.

16. Hori T, Shang WH, Takeuchi K and Fukagawa T. The CCAN recruits CENP-A to the centromere and forms the structural core for kinetochore assembly. J Cell Biol. 2013; 200(1):45-60.
17. Kimura H, Hayashi-Takanaka Y, Goto Y, Takizawa N and Nozaki N. The organization of histone H3 modifications as revealed by a panel of specific monoclonal antibodies. Cell Struct Function. 2008; 33(1):61-73.
